# Supplementary figures and images for: Vaccinia virus D10 has broad decapping activity that is regulated by mRNA splicing
Source: PLoS Pathog. 2022 Feb 24;18(2):e1010099. doi: 10.1371/journal.ppat.1010099 (PMC8903303; doi:10.1371/journal.ppat.1010099)

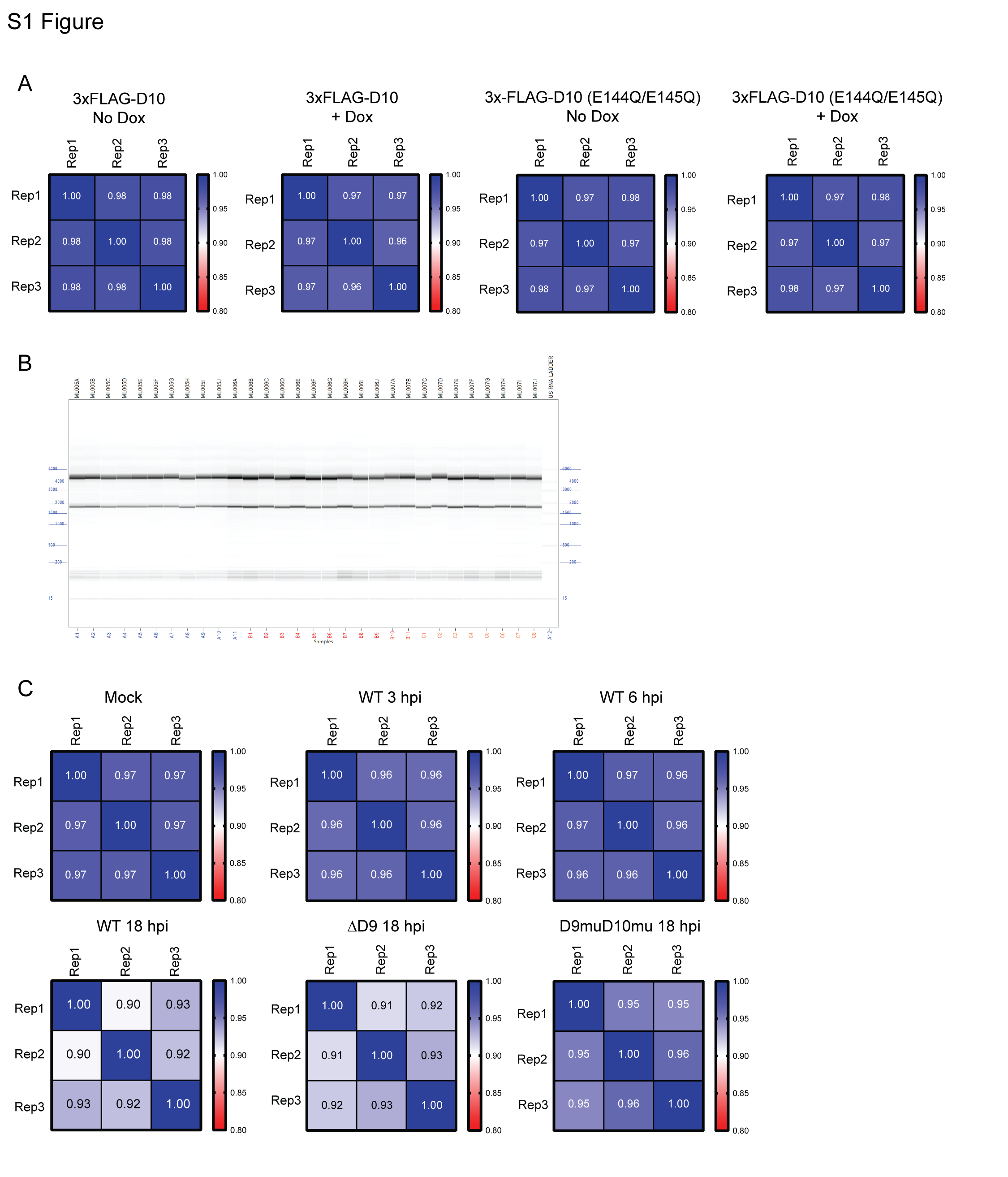

Supplement: S1 Fig — (A) Spearman correlation coefficient between biological replicates from RNA-seq experiment in dox-inducible cells. (B) Bioanalyzer trace results of the RNA samples from VACV-infected HEK293T cells. (C) Spearman correlation coefficient between biological replicates from RNA-seq in VACV-infected HEK293T cells. (TIF) [file ppat.1010099.s001.tif]

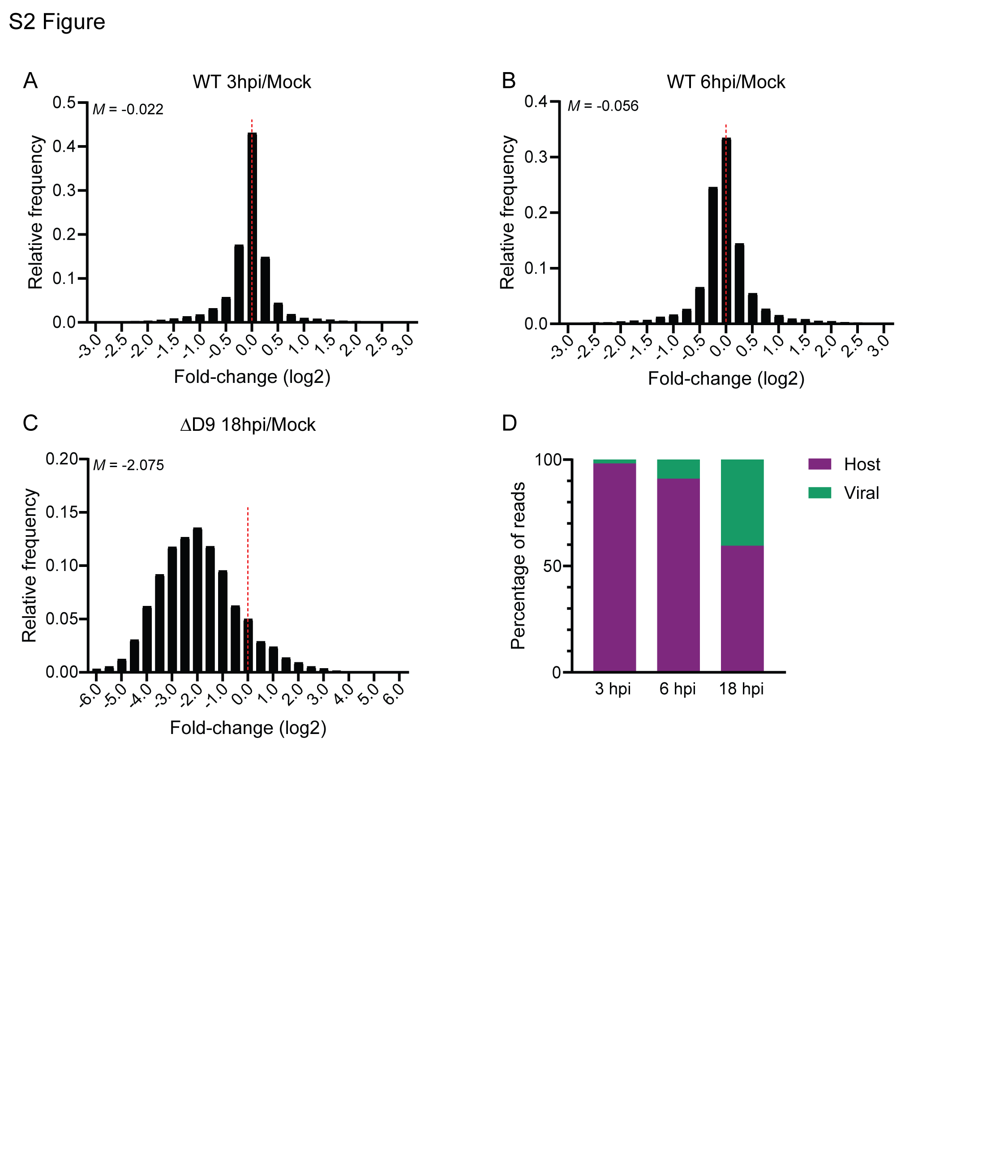

Supplement: S2 Fig — (A-B) The fold-change value for each host transcript was calculated by dividing its expression in WT VACV by its expression in mock infected cells at 3 hpi (A) or 6 hpi (B). The frequency distribution for these values is plotted on each histogram. The dotted red line marks the fold-change value of zero, and the median value (M) is shown for each plot. (C) The fold-change value for each host transcript was calculated by dividing its expression in ΔD9 VACV by its expression in mock infected cells at 18 hpi. The frequency distribution for these values is plotted on the histogram. The dotted red line marks the fold-change value of zero, and the median value (M) is shown for each plot. (D) Percentage of reads mapping to the viral or host genome during WT VACV infection at 3, 6, or 18 hpi. (TIF) [file ppat.1010099.s002.tif]

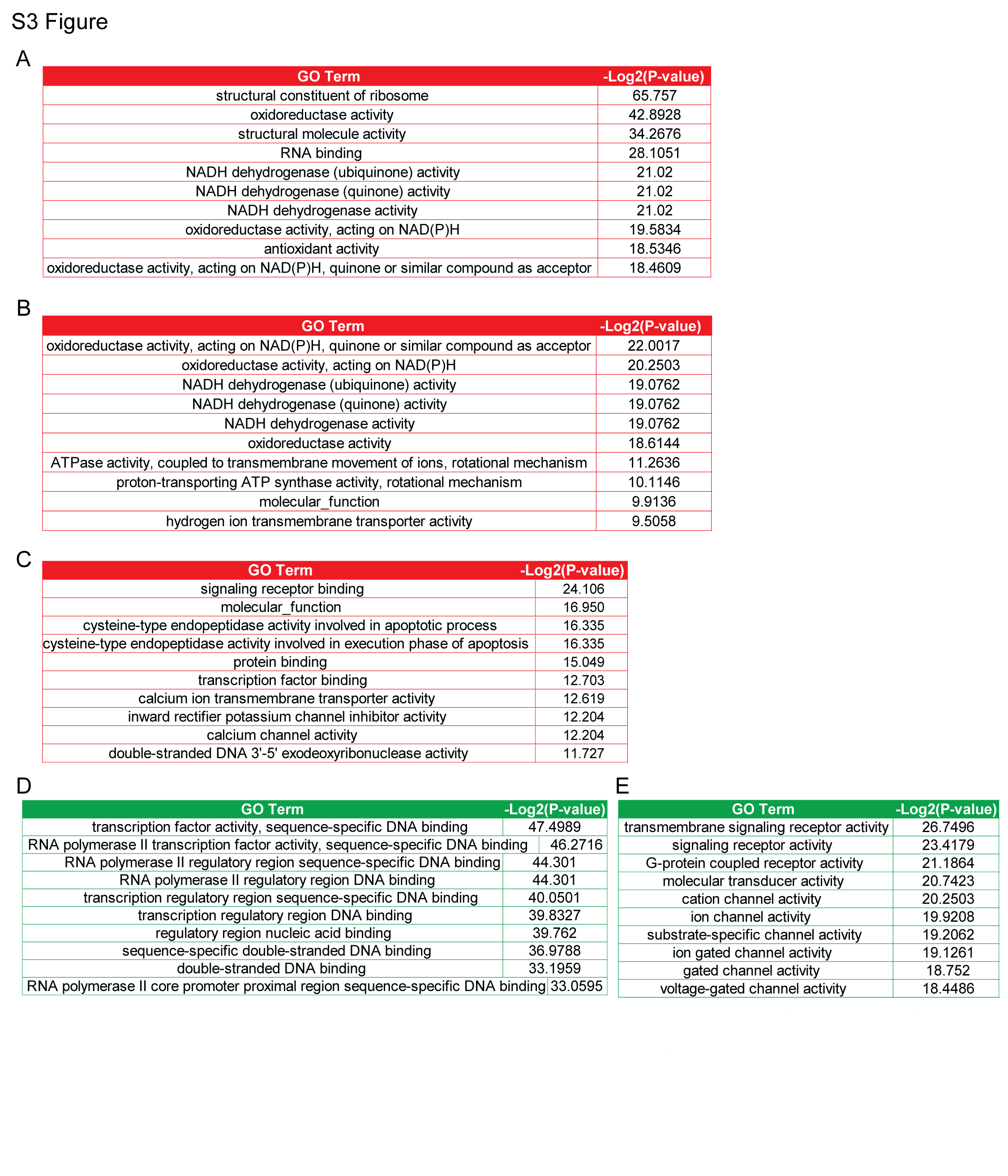

Supplement: S3 Fig — (A-C) GO term analysis for Molecular Function ontology among ranked list of genes downregulated by D10 in dox-inducible cells (A), downregulated by WT VACV infection at 18 hpi relative to mock (B), or downregulated specifically by D10 during VACV infection at 18 hpi (C). Based on their P-values, the top 10 enriched GO terms are shown. (D-E) GO term analysis for Molecular Function ontology among ranked list of genes upregulated by D10 in dox-inducible cells (D) or upregulated by WT VACV infection at 18 hpi relative to mock (E). Based on their P-values, the top 10 enriched GO terms are shown. (TIF) [file ppat.1010099.s003.tif]

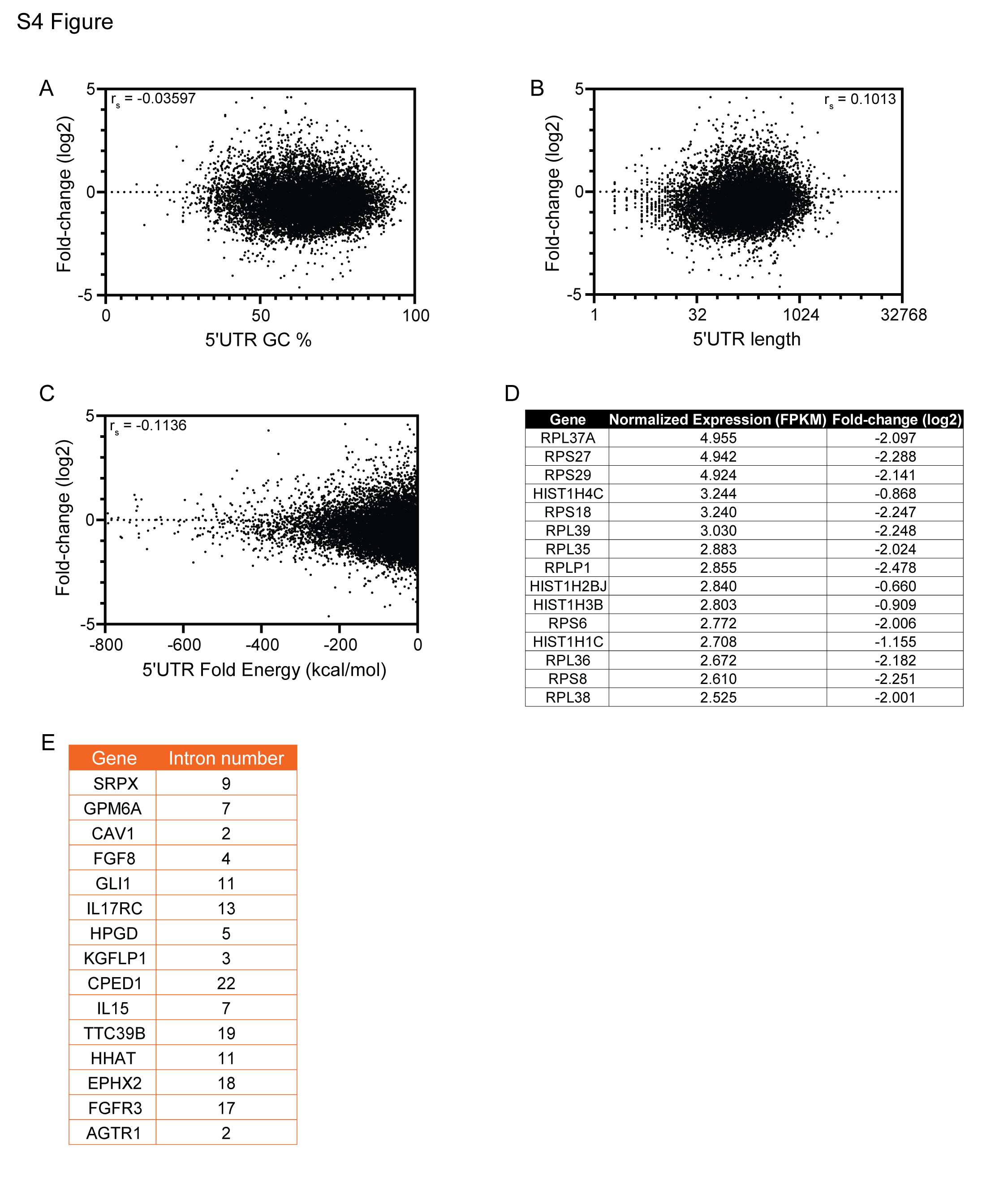

Supplement: S4 Fig — (A-C) Correlation between 5’UTR GC % (A), length (B), or folding energy (C) and fold-change of cellular transcripts upon D10 induction. The Spearman correlation coefficient (rs) is shown. For (A) and (B), there are 78 data points outside the axis limits out of a total of 14,967 data points. For (C), there are 40 data points outside the axis limits out of a total of 12,357 data points. (D) Cellular transcripts were sorted by high expression level then sequentially by low fold-change value upon D10 induction, with the table showing the top 15 genes in this category. (E) Table of top 15 genes most downregulated by D10 during infection, reflected by comparing host transcript abundance in cells infected with the ΔD9 and D9muD10mu strains at 18 hpi, and their corresponding number of introns. (TIF) [file ppat.1010099.s004.tif]

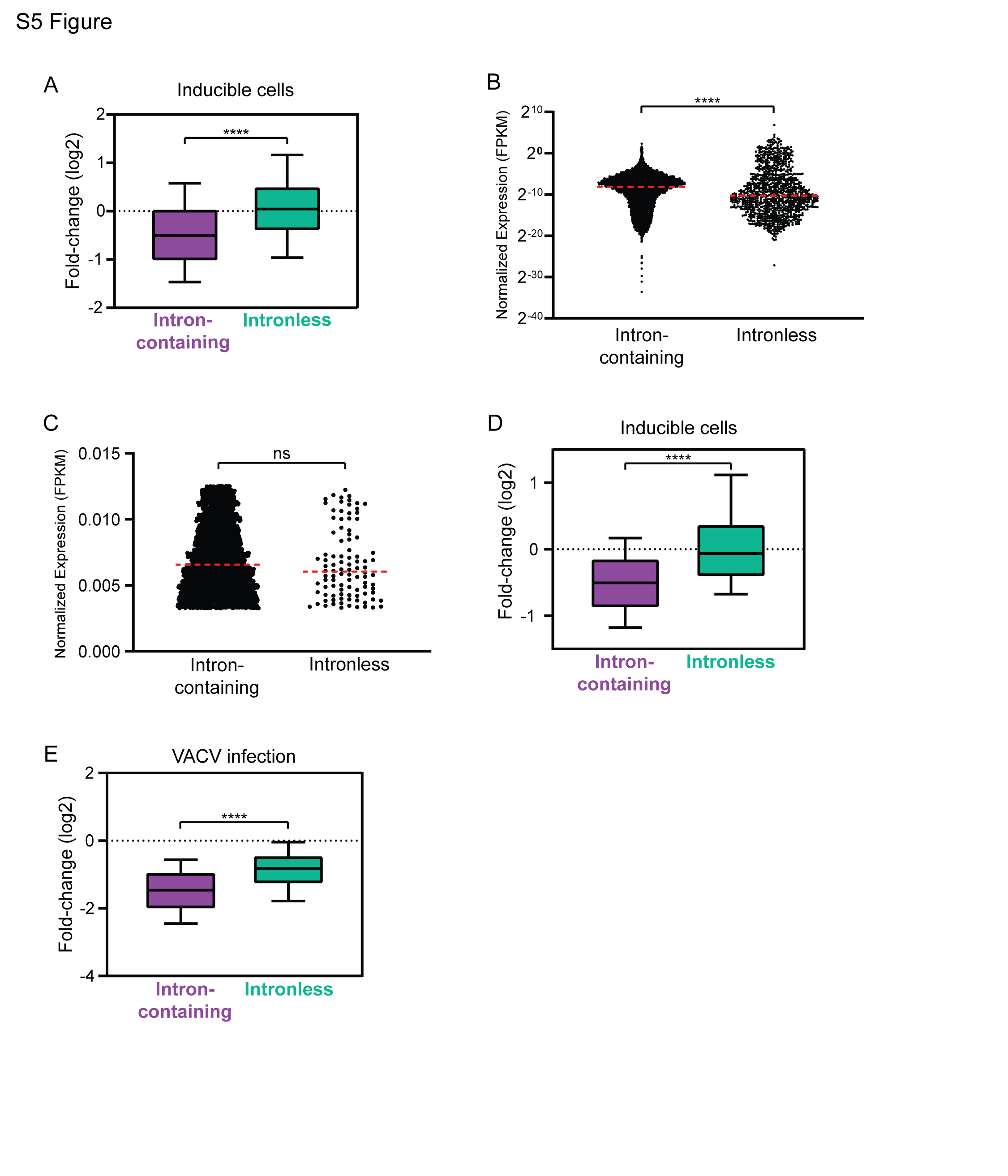

Supplement: S5 Fig — (A) Cellular transcripts were divided into intron-containing or intronless category and plotted against their fold-change value upon D10 induction. ****P≤0.0001, Mann-Whitney test. The whiskers represent the 10th and 90th percentiles. (B) Transcript abundance of intron-containing and intronless genes from (A) was compared. ****P≤0.0001, Mann-Whitney test. The dotted red line represents the median value. (C) Transcript abundance of expression-matched dataset, which consists only the subset of genes expressed within 50th to 75th percentile range. ns-not significant, Mann-Whitney test. The dotted red line represents the median value. (D-E) Cellular transcripts from expression-matched dataset were divided into intron-containing or intronless category and plotted against their fold-change value reflecting D10 activity in dox-inducible cells (D) or during infection (E). ****P≤0.0001, Mann-Whitney test. The whiskers represent the 10th and 90th percentiles. (TIF) [file ppat.1010099.s005.tif]

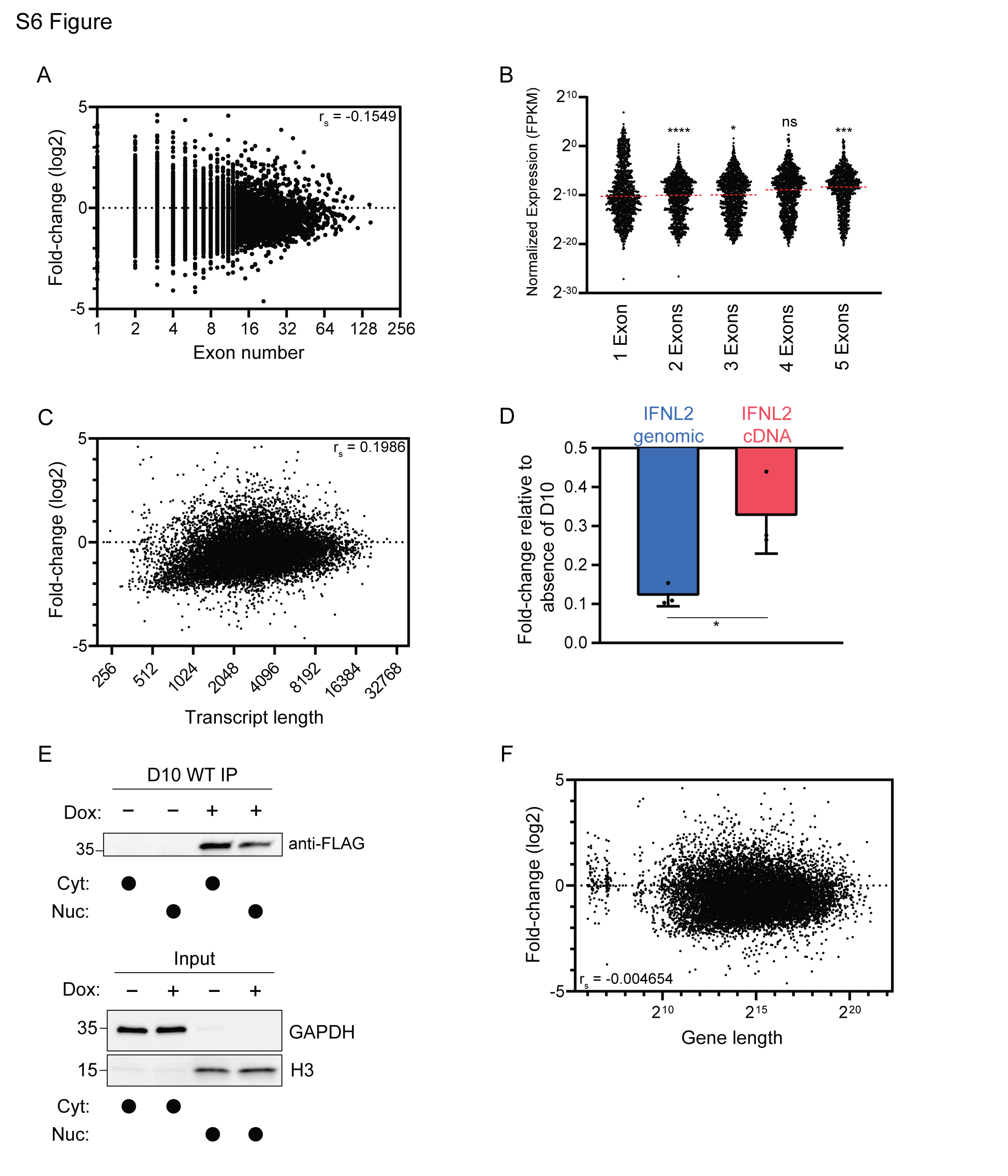

Supplement: S6 Fig — (A) Correlation between exon number and fold-change of cellular transcripts upon D10 induction. The Spearman correlation coefficient (rs) is shown. Out of 13,841 data points, there are 36 data points outside the axis limits. (B) Transcript abundance of cellular transcripts binned according to the number of exons they contain. ns-not significant, *P≤0.05, ***P≤0.001, ****P≤0.0001, Kruskal-Wallis test followed by Dunn’s multiple comparison test versus “1 Exon”. The dotted red line represents the median value. (C) Correlation between transcript length and fold-change of cellular transcripts upon D10 induction. The Spearman correlation coefficient (rs) is shown. There are 271 data points outside the axis limits out of a total of 15,593 data points. (D) HEK293T cells were co-transfected with D10, B2 SINE, and either the genomic (5 introns) or intronless cDNA version of the IFNL2 reporter. RT-qPCR was used to quantify levels of IFNL2, which were normalized to B2 SINE, and the fold-change was calculated relative to absence of D10. Each point represents an independent replicate (N = 3). *P≤0.05, unpaired t-test. The bars represent the mean value of the replicates and error bars represent standard deviation. (E) 3xFLAG-D10 WT HEK293T stable cells were either uninduced (-) or dox-induced (+) then fractionated into nuclear and cytoplasmic compartments. The levels of 3xFLAG-D10 in each compartment were visualized by western blotting following immunoprecipitation with anti-FLAG beads, while the levels of Histone H3 (nuclear marker) and GAPDH (cytoplasmic marker) in each compartment were detected in the input samples. (F) Correlation between gene length and fold-change of cellular transcripts upon D10 induction. The Spearman correlation coefficient (rs) is shown. There are 81 data points outside the axis limits out of a total of 15,573 data points. (TIF) [file ppat.1010099.s006.tif]

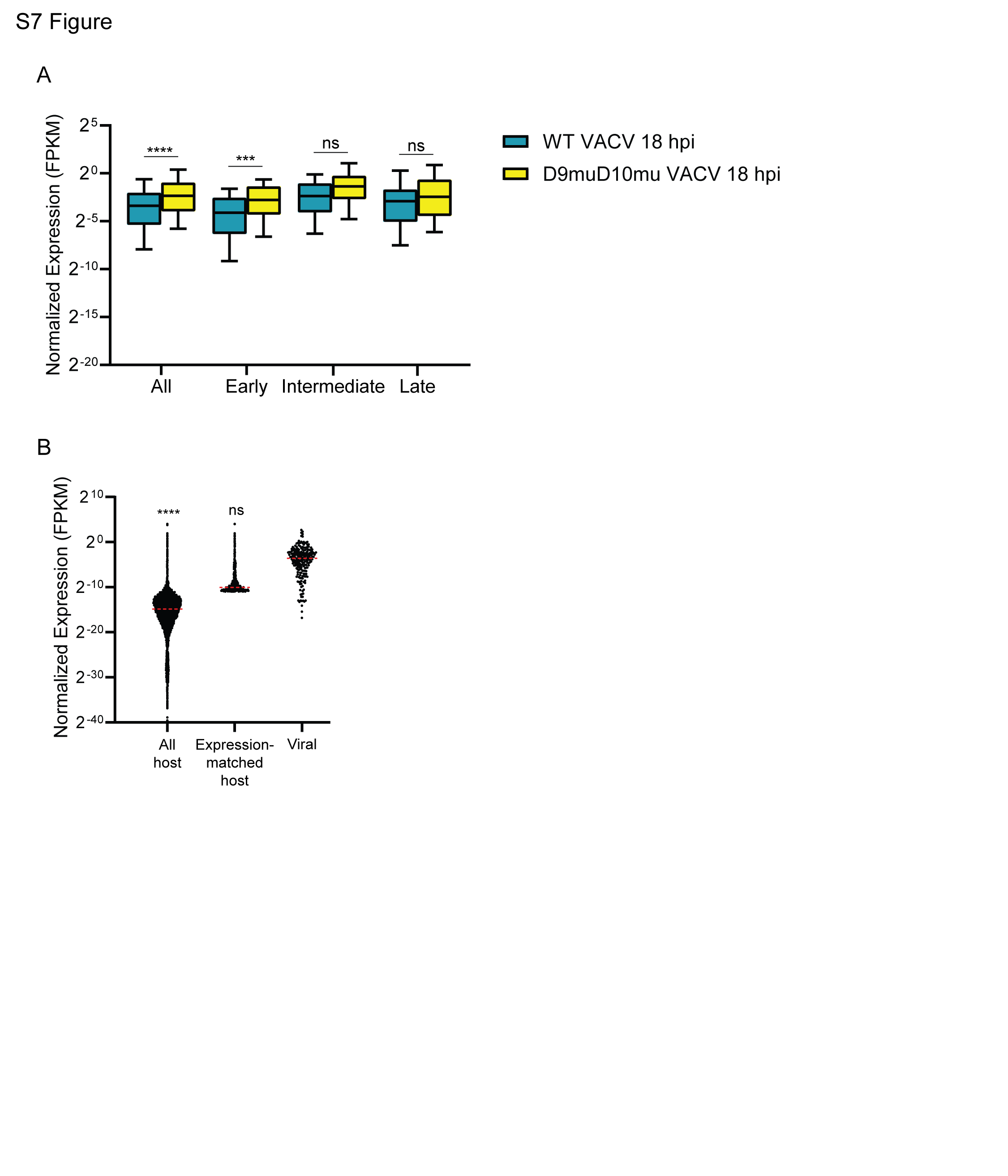

Supplement: S7 Fig — (A) The abundance of viral transcripts from different kinetic classes was compared between WT VACV and D9muD10mu VACV at 18 hpi. ns-not significant, ***P≤0.001, ****P≤0.0001, Kruskal-Wallis test followed by Dunn’s multiple comparison test versus “WT VACV” infection. The whiskers represent the 10th and 90th percentiles. (B) Transcript abundance of viral transcripts, host transcripts, and a subset of expression-matched host transcripts, defined as highly expressed transcripts within the 90th-100th percentile of expression. ns-not significant, ****P≤0.0001, Kruskal-Wallis test followed by Dunn’s multiple comparison test versus “Viral”. The dotted red line represents the median value. (TIF) [file ppat.1010099.s007.tif]
