# Supplementary material for: Vaccinia virus D10 has broad decapping activity that is regulated by mRNA splicing
Source: PLoS Pathog. 2022 Feb 24;18(2):e1010099. doi: 10.1371/journal.ppat.1010099 (PMC8903303; doi:10.1371/journal.ppat.1010099)
Supplement: Attachment — Submitted filename: Response_to_reviewers.pdf [file ppat.1010099.s011.pdf]

Response to reviewer comments

We thank the reviewers for their careful assessment of our manuscript. We have now addressed each of the concerns raised, including through the inclusion of new experiments and a deeper analysis of our existing genomics data. Our responses to each point are detailed below.

Part I - Summary

<u>Reviewer #1:</u> *Many viruses possess the capacity to manipulate the 5' end of host and viral mRNAs to facilitate propagation. The Vaccinia virus (VACV) encode two proteins, D9 and D10, that function to remove the 5' protective m7G cap (decapping) from mRNA. Decapping and degradation of host cell mRNAs is an important strategy to minimize translation from host-cell encoded mRNAs and enhance viral protein translation. In this manuscript the Glaunsinger lab utilizes RNA-seq from Wt VACV,D9 and/or D10 mutant VACA virus infected mammalian cells to identify viral and host cell RNAs targeted by the D9 and D10 decapping proteins. D9 and D10 both target a subset of viral RNAs at a different stage of viral infection. The authors uncover a very interesting observation that D10 predominantly targets host cell mRNAs encoded by intron-containing genes providing a mechanism to preferentially deplete predominantly intron containing host mRNAs while retaining the intronless viral transcripts. This is a significant finding that helps explain the selective advantage that D10 decapping could provide the virus and possibly new insight into the regulation of cellular decapping enzymes. The following are several suggests that would improve the manuscript.*

<u>Reviewer #2:</u> *Ly and colleagues examined the role for the vaccina virus D10 decapping enzyme in the viral infectious cycle. It was found that D10 decaps both viral and cellular transcripts. However, D10 prefers intron-containing transcripts, allowing the intron-less viral transcripts to escape from degradation. The results are supported by excellent genetic and computational studies that reveal a novel link between decapping and splicing in modulating virus-host interactions.*

<u>Reviewer #3:</u> *In this work, Li et al. examine the effect of viral VACV D10 mRNA decapping enzyme on cellular and viral transcripts using RNA-Seq analysis. The authors use two different approaches to examine the effect of D10 on cellular transcripts: 1) through inducible expression of VACV D10 in human cells and 2) during VACV infection. The data show that D10 has a profound effect on cellular mRNA levels, particularly on mRNAs derived from genes containing introns, a feature notably absent from VACV genes. The authors also present data regarding the effect of VACV D10 and D9 on viral transcript levels.*

Overall, the paper presents a novel and intriguing intron-based model for viral mRNA decapping enzymes to preferentially target host transcripts as opposed to viral mRNAs. The specific targeting of host mRNAs could promote viral mRNA access to the ribosomes, along with the shutdown of host protein synthesis.

Part II – Major Issues: Key Experiments Required for Acceptance

<u>Reviewer #1:</u> *(No Response)*

<u>Reviewer #2:</u> *1. D10 is expressed late in viral infection. Why would decapping of cellular transcripts be beneficial for viral gene expression when, for example, ribosomes are needed early during infection?*

Ribosomes are needed throughout infection, but we believe that the ability of VACV to shift ribosome access towards viral mRNA is most crucial late in infection, as this is when the abundance of viral transcripts is generally highest. In support of this idea, we analyzed the fraction of reads mapping to the viral and host genome and found that viral transcripts only account for 2% and 9% of total reads at 3 hpi and 6 hpi, respectively (new Fig S2D). However, viral transcripts account for 40% of total reads at 18 hpi. Therefore, the expression of D10 late during infection is advantageous for the virus as this represents a period when ribosomes are needed to translate the transcriptome that is dominated by viral transcripts. We described this on lines 153-156 of the manuscript.

2. Does D10 locate to the nucleus?

We addressed this question by fractionating cell lysate and performing immunoprecipitation against D10 protein (new Fig S6E). We detected D10 in both the cytoplasmic and nuclear compartments, indicating that D10 is present in the nucleus. We described this on lines 281-285 of the manuscript.

3. The authors measure RNA abundances throughout their studies and link them to degradation. Could it be that transcription is affected as well by D10? Studies in XRN1-depleted cells could clarify this point.

We agree that a reduction in RNA abundance could be due to increased degradation and/or reduced transcription. As per your suggestion, we performed reporter assays in XRN1-knockout HEK293T cells to assess if the decrease in RNA abundance by D10 is a consequence of increased RNA degradation (new Fig 3D). We observed significant recovery in the expression of the reporter in XRN1-knockout background relative to control background, suggesting that the reduction in RNA abundance can be attributed to cytoplasmic mRNA decay. We clarified this on lines 277-280.

<u>Reviewer #3:</u> *1. The authors provide evidence that mRNAs expressed from genes containing introns are preferentially targeted by VACV D10 for decapping and subsequent degradation. Since this is a novel mechanism for viral-mediated host mRNA decapping, additional supporting evidence would be helpful. Furthermore, since VACV is a cytoplasmic virus, and D10 localization is primarily cytoplasmic/mitochondrial, it is*

hard to envision a model in which cellular cytoplasmic, spliced mRNAs could be targeted once the exon junction complexes are removed. Of course, this does not preclude the possibility of splicing influencing VACV mRNA decapping, but additional evidence would make the claim more convincing.

One way this could be accomplished is through discussion of specific mRNAs most targeted by VACV D10 and their corresponding gene structure. For example, similar to Figure S4C, a table could be provided indicating the mRNAs most affected by D10, along with the corresponding intron architecture of the gene encoding it. These data would be particularly interesting in the context of VACV infection. Furthermore, for Figure 1B, it would be helpful to know the number of introns for ACTB, GAPDH, EEF1A1, and the same would be useful for Figure 3A for ERCC8, GBE1, and GJA1. (Side note: It is fascinating the mRNAs targeted most by VACV function in the mitochondria and during oxidative phosphorylation.)

A second way this could be accomplished would be analysis of additional mRNA species in Figures 3A, C, and D, to ensure that another mRNA feature is not responsible for the targeting by VACV D10.

Lastly, the authors examined whether GC content or 5' UTR length influenced targeting by VACV D10. Other mRNA features could also be ruled out using the RNA-Seq data, such as sequence elements in the 5' or 3' UTR. This seems particularly important since cellular Dcp2 preferentially recognizes mRNAs with a 5' stem-loop structure.

We appreciate your insightful input on our manuscript. We now include data showing that D10 is present both in the nucleus and cytoplasm (see new Figure S6E), and thus could in theory distinguish the splicing status of transcripts in either compartment. As mentioned in the discussion section, we postulate two possible mechanisms by which D10 preferentially targets transcripts derived from intron-containing genes: (i) D10 could interact with the spliceosome machinery and be co-transcriptionally loaded onto mRNAs and (ii) D10 could be recruited to exon-junction complexes and preferentially target transcripts that have been spliced. Because D10 is present in both the nucleus and cytoplasm, these two models are not mutually exclusive, and there may be both nuclear and cytoplasmic attributes that increase a transcript's sensitivity to decapping by D10.

Nonetheless, we agree with your suggestion that additional evidence would bolster the findings already presented in our manuscript. Based on your comments, we added the following analyses to our manuscript:

i. **We added a table (new Table S2) that contains information on the number of introns from the various genes mentioned in our manuscript, including the genes analyzed in Figures 1B and 3A.**
ii. **We analyzed the top 15 host mRNAs most downregulated by D10 during infection and their corresponding intron numbers (new Fig S4E). They all**

contained multiple introns, consistent with D10 preferentially targeting transcripts derived from intron-containing genes. We described this on lines 193-195 of the manuscript.

iii. **To test if structural elements in the 5'UTR could affect D10 targeting, we assessed the relationship between 5'UTR folding energy and D10 targeting efficiency (new Fig S4C). There was no signficant correlation, suggesting that this mRNA feature does not influence D10 targeting. We described this on lines 182-183 of the manuscript.**

iv. **Since transcripts with multiple exons could be longer than ones with a single exon, transcript length could be a confounding variable in our analysis. Thus, we assessed the relationship between transcript length and D10 targeting (new Fig S6C). There was no significant correlation, suggesting that transcript length is not confounding our interpretations of the results. We described this on lines 265-268 of the manuscript.**

v. **Since D10 is present in the nucleus, we examined the relationship between gene length and D10 targeting efficiency (new Fig S6F). As mentioned by reviewer #1 in Part III of these reviews, it is possible that genes with multiple introns are longer, and thus take longer to be transcribed by RNA Pol II. However, there was no significant correlation, suggesting that the transcriptional kinetics of intron-containing genes does not explain our findings. We described this on lines 291-298 of the manuscript.**

2. The dataset describing the difference in viral transcripts in the D9/D10 mutant backgrounds is extremely useful. It would be helpful if more analysis were performed on this dataset to make the data more meaningful, such as the GO Term analysis performed for host genes, along with analysis of 5' poly(A) leader structure of the viral mRNAs in relation to D9/D10 targeting. Also, it would have been useful to analyze the ΔD10 mutant virus to account for effects of D9 alone on viral transcripts, but this experiment could be performed in the future.

Thank you for your suggestions. We agree that further analysis of this dataset would benefit our manuscript, and so we added the following analyses:

i. **As per your suggestion, we performed GO term enrichment analysis on host genes that are most downregulated by D10 during infection (new Fig S3C). We described this on lines 161-170 of the manuscript. In summary, the analysis revealed many apoptosis-related genes being most robustly downregulated by D10 during infection, which was distinct from the GO term enrichment analysis in inducible cells where genes involved in oxidative phosphorylation and mitochondrial function were most downregulated by D10. As described in the manuscript, we believe this is because viral infection (but not D10 expression alone) causes apoptosis and may lead to upregulation of apoptosis-related transcripts that are not as abundant in uninfected cells.**

ii. **One of our findings was that D10 targeted early viral transcripts more strongly than intermediate and late transcripts. To expand on this finding,**

we hypothesized that the abundance of early transcripts would be dramatically altered in the D9muD10mu virus compared to WT virus while the levels of intermediate and late transcripts would stay constant. Indeed, we found that the abundance of early transcripts was significantly upregulated in the D9muD10mu virus whereas the abundance of intermediate and late transcripts was not affected (new Fig S7A). We described this on lines 309-313 of the manuscript.

iii. **To address your question on the possible relationship between 5' poly(A) leader and D9/D10 targeting, we looked into our sequencing reads to check if the 5' poly(A) tract on viral transcripts was sequenced in the RNA-seq experiment. Unfortunately, we could not detect the 5' poly(A) tracts in our sequencing reads, and thus are unable to do a comprehensive analysis on the relationship between 5' poly(A) leader and D9/D10 targeting. Nonetheless, we believe this would be a valuable future analysis, which we elaborated on in the discussion section on lines 397-400.**

Part III – Minor Issues: Editorial and Data Presentation Modifications
Please use this section for editorial suggestions as well as relatively minor modifications of existing data that would enhance clarity.

<u>Reviewer #1:</u> 1. The authors postulate two reasonable mechanisms for the preferential targeting of D10 to introned transcripts in the discussion section. One being a deposition of D10 onto the premRNA by the spliceosome in the nucleus and a second being a recruitment to spliced mRNA through an interaction of D10 with the exon junction complex in the cytoplasm. Although definitively answering how D10 targets intron-containing transcripts is beyond this manuscript, some additional suggestive data would significantly strengthen it. For example, is there any evidence for D10 interactions in protein interactome databases? Does the RNA seq data reveal whether the D10 transcripts were primarily pre-mRNA or mRNA (providing information of what compartment the regulation may occur).

Thank you for your insightful ideas! We agree that looking into protein interaction partners of D10 would be informative and may provide insight into its mechanism of discrimination by intron. Unfortunately, there is no published protein interaction dataset for D10. We hope to explore this research question in future work.

Your idea on assessing whether D10 targets pre-mRNA or mRNA is a great idea, and we calculated the reads mapping to introns in our RNA-seq data, which has been demonstrated to be an accurate readout of pre-mRNA levels (see citation in manuscript). We found that, in the inducible cell lines, D10 WT expression leads to a reduction in exonic read counts, while intronic read counts remain unchanged (Fig 3E). This strongly suggests that D10 robustly downregulates mature mRNAs but does not target pre-mRNAs for degradation. We described this on lines 285-291 of the manuscript. Nevertheless, it is still possible that D10

is actively discriminating transcripts in the nucleus, but not at the pre-mRNA level. We elaborate on this in the discussion section on lines 423-426.

In addition, have the authors considered a kinetic explanation? Does the susceptibility to D10 correlate with pre-mRNA size where longer transcripts (ie multi intron containing transcripts) are retained in the nucleus longer than shorter intronless transcripts and more susceptible to D10?

Since we found that D10 is present in the nucleus (Figure S6E), we considered the kinetic explanation that you described here—specifically, does gene length correlate with D10 targeting efficiency? Similar to your thought, we reasoned that this could be a possibility because longer genes could take longer to be transcribed, and thus be exposed to increased D10 activity in the nucleus. This is particularly important because genes with multiple introns could be longer than intronless genes, which may explain their difference in susceptibility to D10 targeting. However, we did not observe a correlation between gene length and D10 targeting, arguing against a kinetic explanation for the phenotype (Fig S6F). We described this on lines 291-298 of the manuscript.

2. Line 191: A 1.2 fold cutoff is used to evaluate percentage of different transcript that are responsive to D9 or D10. What is the rationale for using 1.2 rather than the more common cutoffs of 2 fold or even 1.5 fold?

We initially used a 1.2-fold cutoff because we found that the effects of D9 and D10 on viral transcripts were quite modest (Figures 4A-B). However, as per your suggestion we have now re-analyzed the dataset using the more standard 1.5-fold cutoff (revised Figure 4C).

<u>Reviewer #2:</u> *Fig. 1A. There is no input signal.*

There is no input signal because the proteins are not highly expressed, and they were only detectable following immunoprecipitation and western blot. We clarified this on lines 102-103.

<u>Reviewer #3:</u> *Introduction:*
1. Line 53: Also increased levels of certain viral mRNAs…
Good point. We modified the sentence to include viral mRNAs

2. Line 62: I suggest addition of semi-colon after "however".
We made adjustments to this sentence to include the semi-colon.

3. Line 71: I suggest Despite "the" broad activity of D10.
Thank you. We made this modification to the sentence.

Results
4. Throughout the results sections, it would be helpful if authors clearly defined the cell line or mutant strain used for each experiment: e.g., 3XFLAG-D10 or 3XFLAG-D10 (E144Q/E145Q) or ΔD9 compared to vD9muD10mu. I also suggest adding these labels to the figures in the paper for increased sample clarity.
We clarified this throughout the manuscript (e.g., lines 162-164, 185-187). We also added labels to Figures 1, 2, and 4 to clarify the dataset used.

5. Line 87: Figure 1B Given later findings, what is exon/intron number for these housekeeping genes?
We've added a table that includes the intron information for all genes we've measured abundance of using qPCR (Table S2).

6. Lines 88-89: Figure 1B Why is 7SK RNA increased when D10 is expressed?
We believe the increase in 7SK RNA could be an indirect effect of D10 expression. For example, D10 could be degrading transcripts encoding for inhibitors of 7SK RNA expression, which would consequently lead to an upregulation of 7SK RNA.

7. Lines 143-145: What is exon/intron structure of other genes besides histone genes (e.g., RPL37A)?
All the other genes on that table are ribosomal proteins, all of which contain at least two introns. The ribosomal protein genes are well targeted by D10, but they ended up on the table because they are highly expressed.

8. Line 154: What is exon/intron structure of ERCC, GBE1, etc.?
We've added a table that includes the intron information for all genes we've measured abundance of using qPCR (Table S2).

Figures
9. Figure 1: Suggest adding labels for C and D cell line D10 protein version expressed (3XFLAG-D10 and 3XFLAG-D10 (E144Q/E145Q) and for E and F mutant strain (e.g., ΔD9 for E) and, hours post-infection—this would be useful for all subsequent figures as mentioned above.

Thank you for your suggestion. We added labels to these figures as well as other figures (Figures 2 and 4) where labels would be helpful.
